# Presynaptic c-Jun N-terminal Kinase 2 regulates NMDA receptor-dependent glutamate release

**DOI:** 10.1038/srep09035

**Published:** 2015-03-12

**Authors:** Robert Nisticò, Fulvio Florenzano, Dalila Mango, Caterina Ferraina, Massimo Grilli, Silvia Di Prisco, Annalisa Nobili, Stefania Saccucci, Marcello D'Amelio, Michela Morbin, Mario Marchi, Nicola B. Mercuri, Roger J. Davis, Anna Pittaluga, Marco Feligioni

**Affiliations:** 1 Laboratory of Pharmacology of Synaptic Plasticity, EBRI “Rita Levi-Montalcini” Foundation, Rome, 00143, Italy; 2 Laboratory of Experimental Neurology, IRCCS Fondazione Santa Lucia, Rome, 00143, Italy; 3 Department of Pharmacy, Pharmacology and Toxicology Section, University of Genoa, Genoa, 16148, Italy; 4 University Campus Biomedico, 00100 Rome; 5 Neuropathology & Neurology V - IRCCS Foundation C. Besta Milan, 20133, Italy; 6 Center of Excellence for Biomedical Research, University of Genoa, Genoa, 16132, Italy; 7 Department of Biochemistry and Molecular Biology, Howard Hughes Medical Institute and Program in Molecular Medicine, University of Massachusetts Medical School, Worcester, Massachusetts, 01605, USA; 8 Department of Physiology and Pharmacology, Sapienza University of Rome, Rome, 00185, Italy

## Abstract

Activation of c-Jun N-terminal kinase (JNK) signaling pathway is a critical step for neuronal death occurring in several neurological conditions. JNKs can be activated via receptor tyrosine kinases, cytokine receptors, G-protein coupled receptors and ligand-gated ion channels, including the NMDA glutamate receptors. While JNK has been generally associated with postsynaptic NMDA receptors, its presynaptic role remains largely unexplored. Here, by means of biochemical, morphological and functional approaches, we demonstrate that JNK and its scaffold protein JIP1 are also expressed at the presynaptic level and that the NMDA-evoked glutamate release is controlled by presynaptic JNK-JIP1 interaction. Moreover, using knockout mice for single JNK isoforms, we proved that JNK2 is the essential isoform in mediating this presynaptic event. Overall the present findings unveil a novel JNK2 localization and function, which is likely to play a role in different physiological and pathological conditions.

Glutamate is the most abundant excitatory neurotransmitter in the brain playing fundamental roles such as neuronal differentiation, migration and survival in the developing brain^1^,^2^, as well as excitatory synaptic transmission and plasticity^3^,^4^. However, in a variety of pathological conditions, including stroke and various neurodegenerative disorders, excessive glutamate release may mediate neuronal injury or death^5^,^6^,^7^. Glutamate exerts its function by activating ionotropic (NMDA, AMPA, kainate) or metabotropic (mGlu) receptors. These receptors are widely distributed in the central nervous system (CNS) and, although they are preferentially located postsynaptically, they are also involved in neurotransmitter release from presynaptic compartments in different neuronal systems^8^. However, existence of glutamate receptors at presynaptic terminals has been suggested by immunochemical localization experiments^9^,^10^, functional studies^11^,^12^ and receptor subunit trafficking analyses^9^,^13^. Physiological NMDA receptor (NMDAR) activity underlies normal synaptic plasticity, whereas its excessive activation leads to apoptosis and excitotoxicity. These events appear to be mediated, among other proteins, by c-Jun N-terminal kinase (JNK) phosphorylation which triggers signaling pathways associated with acute and chronic diseases^14^,^15^,^16^. Three genes are known to encode the different isoforms of JNK^17^: JNK1 and JNK2 that are expressed in a ubiquitous manner, while JNK3 is mainly expressed at neuronal level^18^. Genes associated with JNK expression are essential for mouse development^19^ and JNK isoforms act as key proteins for brain development, including neuronal migration, dendrite formation and axon maintenance, but also neuronal death^20^,^21^,^22^. It has been suggested that following its activation, postsynaptic NMDARs interact with JNK, in fact, JNK inhibition reduces NMDAR-mediated neuronal apoptosis in organotypic hippocampal slice culture^23^. Although JNKs are ubiquitously expressed, there is lack of evidence for their expression and physiological role at presynaptic sites.

In the present study we have investigated, by using biochemical, imaging and functional approaches, the role of JNKs in controlling glutamate release evoked by NMDA.

## Results

### c-Jun N-terminal kinase (JNK) regulates presynaptic glutamate release

**Figure 1:**
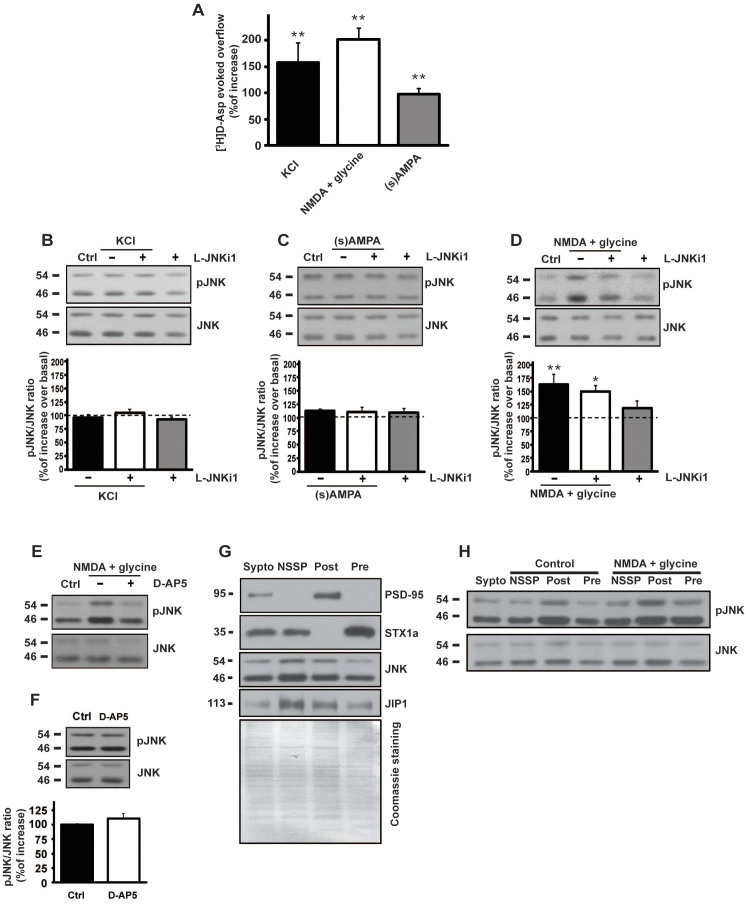
Effect of different pharmacological stimuli on presynaptic glutamate release and biochemical modulation of JNK. (A) Glutamate release evoked by different stimuli. Wild-type mice cortical synaptosomes preloaded with radioactive tracer were incubated with stimuli as indicated. The experiment demonstrated that every stimuli applied were able to induce the release of the neurotransmitter measured as radioactive D-Aspartate. Results are normalized vs. the basal release and expressed as percentage of induced overflow. Means ± s.e.m. *n* = 3 experiments run in triplicate (three superfusion chambers for each experimental condition), **p < 0.01 vs. basal release, Tukey's test. (B–C–D) Cropped WB are representative of the activation of JNK showing P-JNK protein levels upon 90 sec of KCl (8 mM) stimulus (C), 10 min of NMDA (100 μM) + glycine (1 μM) (B), 10 min of (s)AMPA (50 μM) (D) and L-JNKi1 treatment in synaptosomal preparation. The quantifications are expressed as percentage of increase of P-JNK/JNK ratio over control (at 100) and show that only the NMDA + glycine stimulus induced an increase in JNK phosphorylation. Means ± s.e.m. *n* = 4, **p < 0.01 vs 100% of control, Tukey's test. L-JNKi1 does not reduce NMDA effect, *p < 0.05 vs 100% of control, Tukey's test. (E) The NMDA receptor antagonist D-AP5 (50 μM) was incubated in synaptosomal preparation and was able to prevent the JNK phosphorylation induced by NMDA stimulus. Cropped representative WB. (F) D-AP5 (50 μM) was incubated in synaptosomal preparation. Control and D-AP5 conditions not differ in JNK activation. Means ± s.e.m. *n* = 4, Student's test. Cropped representative WB. (G) WB analysis of presynaptic JNK and JIP1. Cropped WB represents the synaptic compartment ultrafractionation of cortical mice synaptosomes. The purity of the preparation has been tested with anti-PSD95 and anti-STX1a as postsynaptic and presynaptic markers. JNK and JIP1 are clearly present also at the presynapse and they can therefore interact together. Lanes loading includes total synaptosomes (Sypto), non-synaptic soluble proteins (NSSP), post-synaptic fraction (Post), pre-synaptic fraction (Pre). Coomassie blue staining has been performed as loading control. H) Ultrasynaptic fractionation of mice cortical synaptosomes was prepared to show presynaptic JNK activation after NMDA stimulus. Cropped representative WB.

Glutamate release experiments, monitored as release of preloaded [3H]D-aspartate ([3H]D-ASP), were performed to investigate the role of JNK kinase in the presynaptic compartment. The radiactive tracer allows to measure glutamate exocytosis from isolated nerve terminals avoiding the problems related to the enzymatic degradation of glutamate^24^,^25^. From here on, throughout the manuscript, the release of preloaded [3H]D-ASP will be referred to as glutamate release. Mouse cortical terminals were tested for glutamate release using different stimuli up-down superfused on a thin layer of synaptosomes, according to a standard procedure^26^. Transient (90 s) exposure of cortical nerve terminals to a mild depolarizing stimulus (8 mM KCl) induced a significant exocytotic-like [^3^H]D-Asp overflow (158 ± 37% vs. control. **p < 0.01), which is comparable to that obtained by 10 min exposure to 100 μM NMDA (202 ± 21% vs. control. **p < 0.01). NMDA stimulus was applied in Mg^2+^-free medium to ensure the operability of the presynaptic NMDA receptor^24^. Similarly, a milder 10 min stimulus of 50 μM (s)-AMPA was able to give a significant [^3^H]D-Asp overflow (98 ± 11% vs. control. **p < 0.01) (fig. 1A). Both stimuli are known to trigger Ca^2+^-dependent neurotransmitter release^27^.

### Biochemical analysis of c-Jun N-terminal Kinase (JNK) activation upon different stimuli in cortical mouse synaptosomes

Among the many intracellular partners activated by the above used stimuli, the involvement of the JNK protein has been investigated in cortical mouse synaptosomes. Synaptic JNK activation, measured as phosphorylated protein induced by different stimuli, was measured by biochemical experiments (fig. 1B–C–D). Only 10 min exposure to NMDA (100 μM) + glycine (1 μM) in Mg^2+^-free medium stimulus induced a significant increase in JNK phosphorylation (+63 ± 18% vs. control = 100%. **p < 0.01). KCl and AMPA left unchanged the level of pJNK respect to the control. The NMDA-evoked release experiments were performed in Mg^2+^-free medium solution to maintain the NMDA receptors active without the Mg^2+^ ‘block’^26^. Notably the NMDA blocker D-AP5 (50 μM) was able to reverse the NMDA-mediated increase in JNK phosphorylation (Fig. 1E). An additional study was carried out to exclude the possibility that the basal release of glutamate reaches a sufficient concentration able to induce NMDA activation in absence of any exogenous stimulus and leading to JNK phosphorylation. The NMDAR antagonist D-AP5 was applied to synaptosomes for 10 min and no changes in phosphorylation of JNK (+10.0 ± 8.9% vs Ctrl 100%) were detected (fig. 1F).

It was demonstrated that postsynaptic NMDAR induces JNK activation through the interaction of JNK scaffolding protein JIP1. In line with this, it was observed that the NMDAR activity in JIP1 KO mice is reduced^28^. Here in order to assess whether also presynaptic NMDAR-signaling pathway might involve the signaling formed by NMDA/JIP1/JNK proteins, we tested for the presence of JNK and JIP1 at the presynaptic level.

For this, we used an ultrastructural fractionation of synaptosomes^9^ and purity of the fractions were analyzed before use. PSD-95 immunoreactivity was observed only at the post-synaptic fraction (Post) while Syntaxin 1a (STX1a) resulted enriched in the presynaptic fraction (Pre) and absent in the Post. The non-synaptic soluble proteins (NSSP) fraction was only contaminated by STX1a which it could be attributed to docking vesicles at the presynaptic membrane (fig. 1G). As expected JNK and its scaffolding protein JIP1 were present at both NSSP and Post fractions. Most importantly they were also clearly present at the presynaptic site (fig. 1G).

To demonstrate that, in synaptosomes, upon NMDA stimulus the activation of JNK is mainly presynaptic, cortical synaptosomes were stimulated and later subjected to ultrasynaptic fractionation. We found that JNK phosphorylation was strongly augmented at the presynaptic site (fig. 1H).

To confirm that JNK activation triggered by NMDA could pass through JNK-JIP1 interaction, we used a specific JNK inhibitor (L-JNKi1) which is able to disrupt the interaction of JNK with its JNK-binding domain (JBD) targets^29^. It is a cell permeable peptide (CPP) composed the JBD amino acid sequence linked to 10–amino acid carrier peptide derived from the HIV-Tat 48–57 sequence^29^. Cortical synaptosomes were treated for 30 min with 2 μM of L-JNKi1 after which the same stimuli above described were applied. In accordance with already published data the time frame and concentration used were enough to let the inhibitor diffuse and inhibit JNK^29^,^30^. In line with the evidences that L-JNKi1 does not affect JNK phosphorylation^31^, pre-incubation of synaptosomes with L-JNKi1 resulted in increased JNK activation when NMDA stimulus was applied (+49 ± 10% vs control = 100%. *p < 0.05). In contrast, basal JNK phosphorylation was unaltered when L-JNKi1 was applied alone (fig. 1B). In the case of KCl and AMPA the application of L-JNKi1 left JNK phosphorylation unaffected (fig. 1C–D). Altogether these results indicate that NMDA stimulus specifically modulates JNK activity.

### L-JNKi1 does not induce changes in vesicle machinery proteins and in NMDA or AMPA receptor subunit levels

We next monitored the expression levels of some presynaptic proteins such as syntaxin 1a (STX1a), synapsin (SYN), MUNC18-1 and synaptotagmin 1 (SYT1) since their alteration compromises the neurotransmitter release efficiency^32^,^33^. In our experimental conditions NMDA and L-JNKi1, when applied alone or in combination, did not modify the expression of STX1a, SYN, MUNC18-1 and SYT1 in synaptosomes (Supplementary Fig. 1A). Similarly, NMDA and AMPA receptor subunits expression (Supplementary Fig. 1B) were unchanged, thus excluding the possibility that NMDA-mediated effect on presynaptic JNK might depend on specific modifications of receptors subunits.

### L-JNKi1 inhibitor modulates presynaptic glutamate release and reduces NMDA-evoked exocytosis of fluorescent dye from synaptosomes

**Figure 2:**
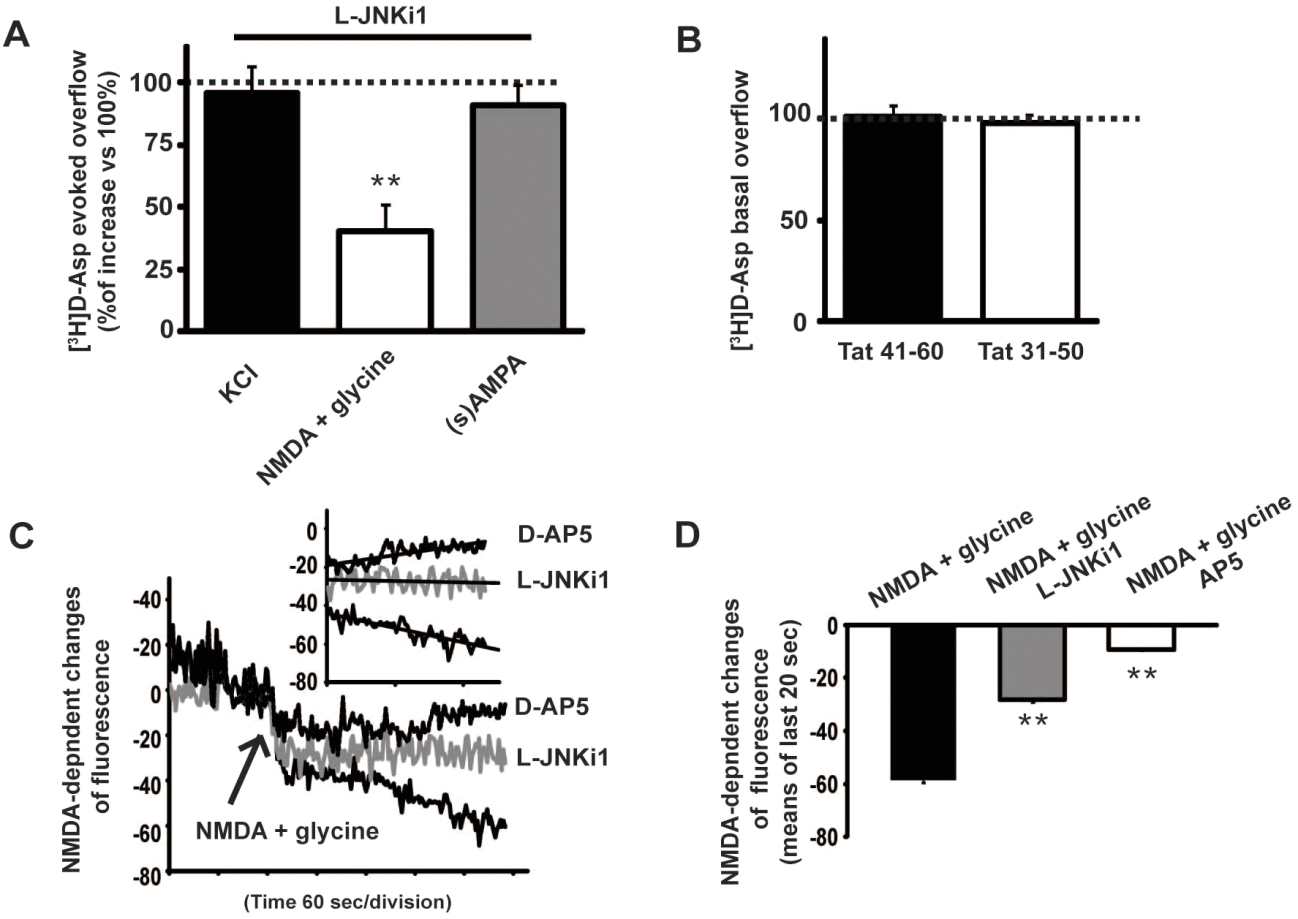
Effect of JNK inhibitor on evoked-glutamate release and fluorescent dye exocytosis. (A) Mice cortical synaptosomes where treated with the JNK inhibitor L-JNKi1 (2 μM) for 30 min before stimuli as indicated. Among the other stimuli, the JNK inhibitor was able to decrease the release of glutamate evoked by NMDA (100 μM) + glycine (1 μM). Results measured as neurotransmitter overflow are normalized vs. the evoked release (at 100) without L-JNKi1. Means ± s.e.m. *n* = 3 experiments run in triplicate (three superfusion chambers for each experimental condition), **p < 0.01 vs 100% of NMDA-evoked release, Tukey's test. (B) Tat 20 mer (41–60) (2 μM) and (31–50) (2 μM) were used as control for the peptide L-JNKi1 which contain a sequence of the Tat protein (48–50) used as cargo for cell permeability. Both the 20 mer contains the cargo sequence and none of them were able to modify the basal release of glutamate in cortical synaptosomes. The synaptosomes loaded with radioactive neurotransmitter were treated with the Tat 20 mer. Results are measured as neurotransmitter overflow are normalized vs the basal release (at 100) without 20 mer Means ± s.e.m. *n* = 3 experiments run in triplicate (three superfusion chambers for each experimental condition), Student's *t*-test (C) Wild-type mice cortical synaptosomes were preloaded with Synaptogreen, a fluorescent dye that is incorporated in the presynaptic release vesicle. Synaptosomes were then incubated, where indicated, with L-JNKi1 30 min before stimuli. Representative traces of Synaptogreen fluorescence decadence are here shown. L-JNKi1 reduced the fluorescence run-down evoked by NMDA (100 μM) + glycine (1 μM). As control the NMDA inhibitor D-AP5(50 μM) has been also used. To better appreciate the effect of the inhibitor traces of the last 2 min of the experiment have been graphed in the little panel. (D) Quantification of the fluorescence decadence upon NMDA + glycine stimulus. Data are calculated as mean of the last 20 sec of the experiments. Means ± s.e.m. *n* = 4, **p < 0.01 vs NMDA alone, Student's *t*-test.

Interestingly, among the 3 stimuli used, only the NMDA-stimulated glutamate release was strongly inhibited (−60 ± 10% vs. control = 100%. *t*-test, **p < 0.01) by L-JNKi1 (fig. 2A), indicating a specificity of the JNK pathway in modulating only the NMDA-evoked glutamate release. In order to exclude that the effect measured was due to the Tat sequence contained in the L-JNKi1, Tat peptides fragments, enclosing amino acids 48–57, were applied on synaptosomes. Nor Tat 41–60 (100.85 ± 5.18% vs. control = 100%; *t*-test) neither Tat 31–50 (97.65 ± 4.11% vs. control = 100%; *t*-test) were able to decrease the glutamate spontaneous release (fig. 2B).

The capability of JNK inhibition to reduce the effect of presynaptic NMDA was also investigated by measuring the vesicle exocytosis. The fluorescent dye (Synaptogreen), which is incorporated into presynaptic vesicles, is released into the extracellular space losing its fluorescence. The fluorescence decrease reflects the exocytosis extent. The NMDA-evoked exocytosis was monitored by spectrofluorophotometer measurement. Synaptosomes, pre-loaded with the fluorescent dye, were exposed to NMDA and glycine stimulus, as control, or with a pretreatment for 30 min with L-JNKi1 or D-AP5. The decrease in fluorescence intensity due to the released dye was registered for 6 min in the two preparations (fig. 2C). As expected, NMDA (100 μM) + glycine (1 μM) stimulus induced a reduction in fluorescence (−58.7 ± 0.9%) which it was reduced by L-JNKi1 application (−28.1 ± 1.1% vs. NMDA + glycine. *t*-test **p < 0.01) and more stronger with D-AP5 that was used as control since is an NMDA channel blocker (−9.2 ± 1.3% vs. NMDA + glycine. *t*-test **p < 0.01), (fig. 2D).

### Time-lapse imaging recording of the stimulated exocytosis from synaptosomes

**Figure 3:**
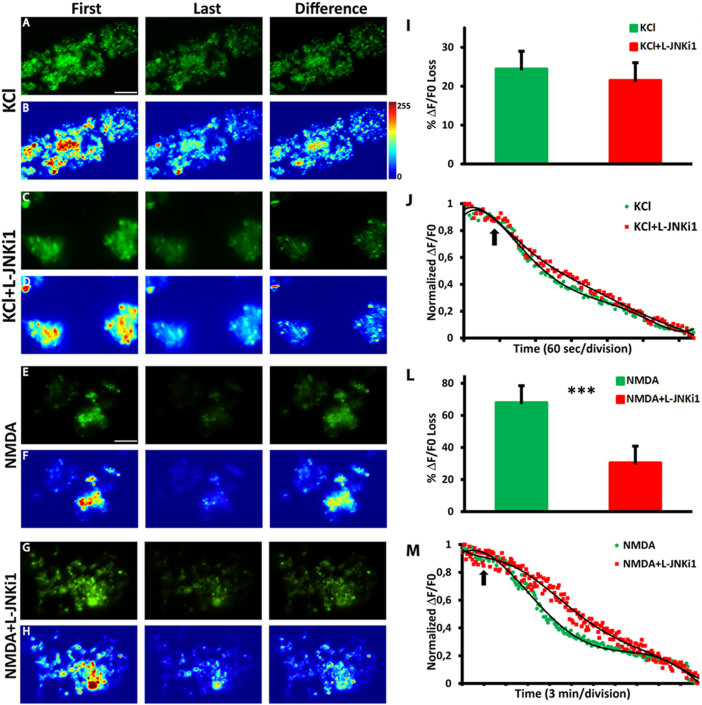
L-JNKi1 pretreatment does not affect KCl responses but reduces NMDA stimulated exocytosis. Representative images of Synaptogreen (FM 1-43) stained cortical synaptosomes stimulated after 1 min with KCl alone (A) or pretreated with L-JNKi1 (C) and recorded for 6 min; or stimulated after 4 min with NMDA alone (E) or pretreated with L-JNKi1 (G) and recorded for 30 min. First, is the first image of the time-lapse sequence. Last, is the last image of the time-lapse sequence. Difference, is the image obtained by subtracting the last image to the first of the time-lapse sequence. (B,D,F,H) are the corresponding fluorescence intensity color map (Jet Palette) images. Brighter spots correspond to functional presynaptic terminals. (I) Average percentage of fluorescence loss for KCl (green) and KCl + L-JNKi1 (red). (J) Representative traces of destaining time courses for KCl and KCl + L-JNKi1. Arrow indicates the KCl stimulus. (L) Average percentage of fluorescence loss for NMDA + glycine and NMDA + glycine + L-JNKi1. (M) Representative traces of destaining time courses for NMDA + glycine (green) and NMDA + glycine + L-JNKi1 (red). Arrow indicates the NMDA stimulus. Data are derived from four independent experiments. In each experiment three coverslips for each experimental group were analyzed. Scale bar A and J = 10 μm.

Synaptogreen fluorescence dye exocytosis was also studied through video time-lapse recording. This additional technique allows to better appreciate the synaptosomal exocytosis kinetics compared to spectrofluorophotometer. By analyzing the images, it is possible to identify responding synaposomes and to analyze the fluorescence changes of synaptic puncta during the experiment. Administration of 30 mM KCl alone was able to trigger a fluorescence decrease (24 ± 4.57%). Synaptosomes pre-incubation with L-JNKi1 for 30 min before the KCl stimulus evoked a slight lower fluorescence decrease (21 ± 6.21%) which was not significatively different to the KCl alone group (fig. 3A–D, I–J.). In line with spectrofluorophotometer experiments, pre-incubation with L-JNKi1 for 30 min before the NMDA (100 μM) + glycine (1 μM) (Mg^2+^-free medium) stimulus prevented the fluorescence decrease (30.37 ± 9.48%; *t*-test, ***p < 0.001) when compared to the NMDA (67.91 ± 10.57%) stimulated alone group (fig. 3E–H, L–M).

### JNK mediates presynaptic NMDA-dependent glutamate release

**Figure 4:**
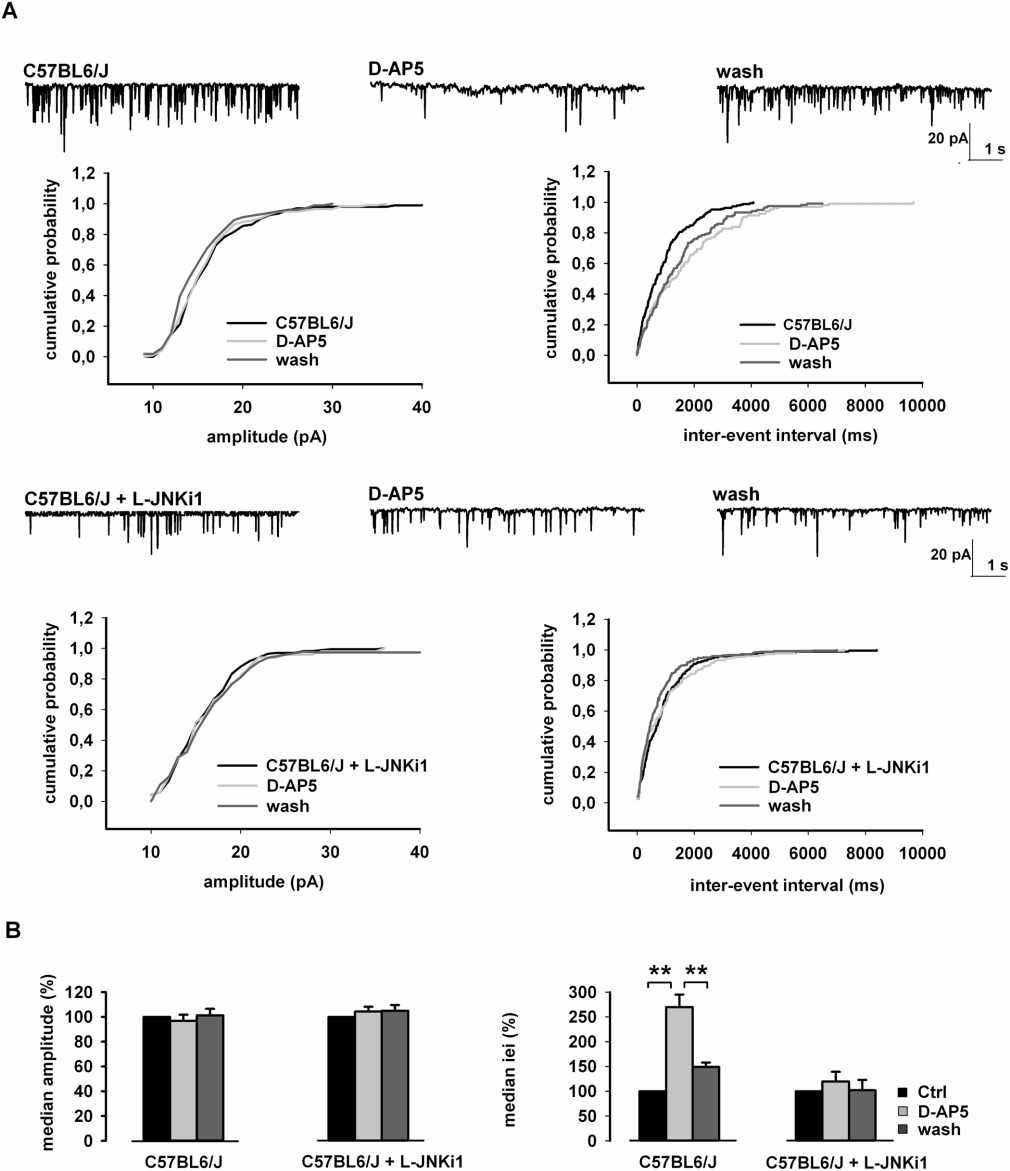
Functional role of JNK in NMDA-dependent glutamate release. (A) Cumulative distributions of mEPSC amplitude (left) and inter-event interval (iei) (right) recorded from a single C57BL6/J neuron, or C57BL6/J neuron pre-incubated with L-JNKi1 in response to D-AP5. The traces on top are obtained from the same neuron, in control conditions, during D-AP5 application and at D-AP5 washout. (B) Histograms of the median values expressed as percentage versus control (100%) of mEPSC amplitude (left) and iei (right) recorded from 11 neurons of C57BL6/J, and 10 neurons of C57BL6/J pre-incubated with L-JNKi1 (n = 10) in response to D-AP5. **p < 0.01 (t-test).

Glutamate spontaneously released in the synaptic cleft can in turn activate the NMDARs located at the presynaptic terminals thus allowing a further tonic facilitation of glutamate release^34^. Here we performed electrophysiological recordings to investigate the functional role of JNK in glutamate release. We studied the effect of L-JNKi1 on NMDA-dependent presynaptic glutamate release measuring miniature excitatory postsynaptic currents (mEPSCs), according to a protocol previously described^34^. The application of D-AP5 in the presence of extracellular tetrodotoxin (1 μM) and picrotoxin (100 μM), and with MK-801 (10 μM) in the patch-pipette (in order to block NMDA channels in the cell we were recording from), induced a reversible increase of the cumulative inter-event interval (iei) distribution (***p < 0.001, K-S test, C57BL6/J vs. D-AP5, n = 11; fig. 4A) and of their median values (**p < 0.01, Student's t-test, ctrl vs. D-AP5, n = 11; fig. 4B), whilst the cumulative and median amplitude of the same events were unaffected (*p* > 0.05, K-S test, C57BL6/J vs. D-AP5, n = 11; fig. 4A; *p* > 0.05 Student t-test, ctrl vs. D-AP5, n = 11; fig. 4B). The reduction in frequency by D-AP5 is due to the blockade of presynaptic NMDA receptors which facilitate tonic glutamate release. In slices pre-incubated with L-JNKi1 the effect of D-AP5 was lost. In fact we found that perfusion with D-AP5 did not lead to significant changes of inter-event interval (*p* > 0.05, K-S test, C57BL6/J + L-JNKi1 vs. D-AP5, n = 10; fig. 4A; *p* > 0.05, Student's t-test, ctrl vs. D-AP5, n = 10; fig. 4B) and amplitude (*p* > 0.05, K-S test, C57BL6/J + L-JNKi1 vs. D-AP5, n = 10; fig. 4A; *p* > 0.05, Student's t-test, ctrl vs. D-AP5, n = 10; fig. 4B). Overall these electrophysiological results indicate that JNK inhibition reduces glutamate release mediated by presynaptic NMDA receptors, suggesting that JNK plays a functional role in NMDA-dependent glutamate release in acute brain slices.

### NMDA does not facilitate glutamate release and JNK phosphorylation in JNK2 knock-out mice

**Figure 5:**
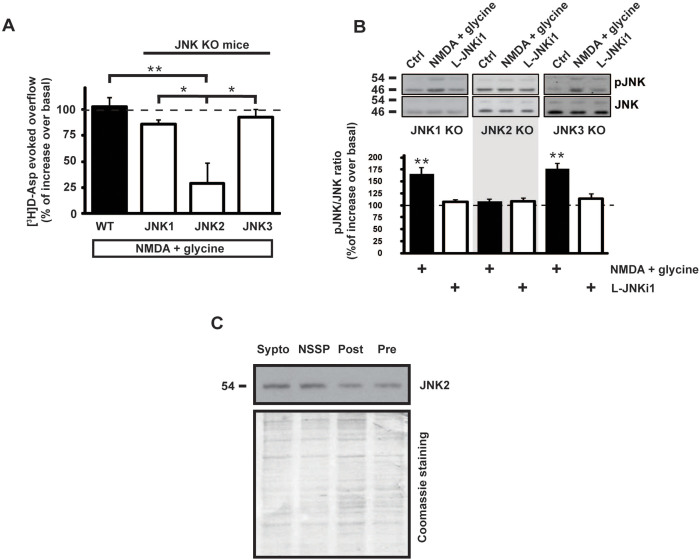
NMDA-evoked glutamate release and NMDA-induced JNK phosphorylation are inhibited in JNK2 KO. (A) NMDA stimulus was used to evoke glutamate release from wild-type and JNK KO mice cortical synaptosomes that were preloaded with radioactive tracer. In synaptosomes from JNK2 KO mice the effect of NMDA (100 μM) + glycine (1 μM) is inhibited indicating that JNK2 presynaptic protein is the responsible of this mechanism. Results are presented as percent of glutamate overflow released under NMDA stimulus compared to the base line of each condition. Means ± s.e.m., *n* = 4 experiments run in triplicate (three superfusion chambers for each experimental condition). **p < 0.01 vs WT; *p < 0.05 vs JNK1 KO or JNK3 KO, Tukey's test. (B) Synaptosomes from cortex of JNK KO mice were stimulated with NMDA (100 μM) + glycine (1 μM). The pJNK/JNK ratio has been evaluated. Representative cropped WB with relative quantification showing pJNK/JNK ratio in JNK1 KO, JNK2 KO or JNK3 KO mice during treatments are reported as shown in blots. Results are expressed as percentage versus control (at 100). Means ± s.e.m., *n* = 3,**p < 0.01 vs 100% of control, Tukey's test. (C) Cortical synaptosomes synaptic fractionation was prepared from wild-type mice to show that JNK2 is also presynaptic located. Lanes loading includes total synaptosomes (Sypto), non-synaptic soluble proteins (NSSP), post-synaptic fraction (Post), pre-synaptic fraction (Pre). Cropped representative WB.

To identify which JNK isoform is activated by NMDA stimulus in presynaptic compartment, glutamate release experiments were conducted on cortical synaptosomes obtained from JNK knock-out (KO) mice. Glutamate release, measured as [^3^H]D-Asp efflux, was evoked by NMDA for 10 min. Interestingly, NMDA evoked release was significantly lower (28 ± 19% over basal overflow; n = 4 Newman-Keuls, **p < 0.01) only in JNK2 KO mice compared to JNK1 and JNK3 KO and wild-type (WT) mice (fig. 5A). This result suggests that presynaptic JNK2 specifically controls NMDA-evoked release.

In order to corroborate the notions indicating a specific involvement of JNK2 in the presynaptic NMDA-evoked glutamate release, we investigated whether the absence of JNK2 reduced JNK phosphorylation. The JNK KO mice used present a deletion of a specific JNK gene, therefore the other remaining JNK proteins are still detectable. To appreciate the different protein expression, JNK immunoreactivity in JNK KO mice has then been analyzed. As already reported^35^ JNK proteins are present as double isoforms (54 kDa and 46 kDa bands) and at around 46 kDa JNK2 shows a faint band up to the JNK1 one. In JNK1 KO mice the JNK1 46 kDa band disappeared, while the JNK2 46 kDa band is lacking in JNK2 KO. In both JNK2 and JNK3 KO mice the 56 kDa bands was found to be reduced (fig. S1C).

Synaptosomes prepared from cortex of JNK-KO mouse were subjected to NMDA stimulus and, as expected, the phosphorylation of JNK induced by NMDA application was totally abolished in JNK2 KO mice as compared with control. In contrast, it was still maintained in JNK1 KO and in JNK3 KO mice where NMDA induced an increase of pJNK immunoreactivity (+65 ± 13% over control = 100%; n = 5 *t*-test **p < 0.01) (+76 ± 10% over control = 100%; n = 4 *t*-test **p < 0.01) (fig. 5B) which is totally comparable with the one obtained in WT mice (fig. 1B). Interestingly NMDA stimulus increased both bands (46 and 54 kDa) of JNK phosphorylation in JNK1 and JNK3 KO mice. This might be due to the fact that JNK2, which is present in both JNK1 and JNK3 KO animals, has two splicing variants at 46 and 54 kDa^36^. In all cases, application of L-JNKi1 alone did not affect JNK phosphorylation, suggesting that JNK functionality in both KO mice is not perturbed in the presence of JNK inhibitor, as also reported for wild-type mice (fig. 5A). The presence of JNK2 at the presynaptic site was then detected by WB on an ultrasynaptic fractionation of mice cortical synaptosomes (fig. 5C).

### Ultrastructural characterization of synaptosomes and localization of JNK2 protein

**Figure 6:**
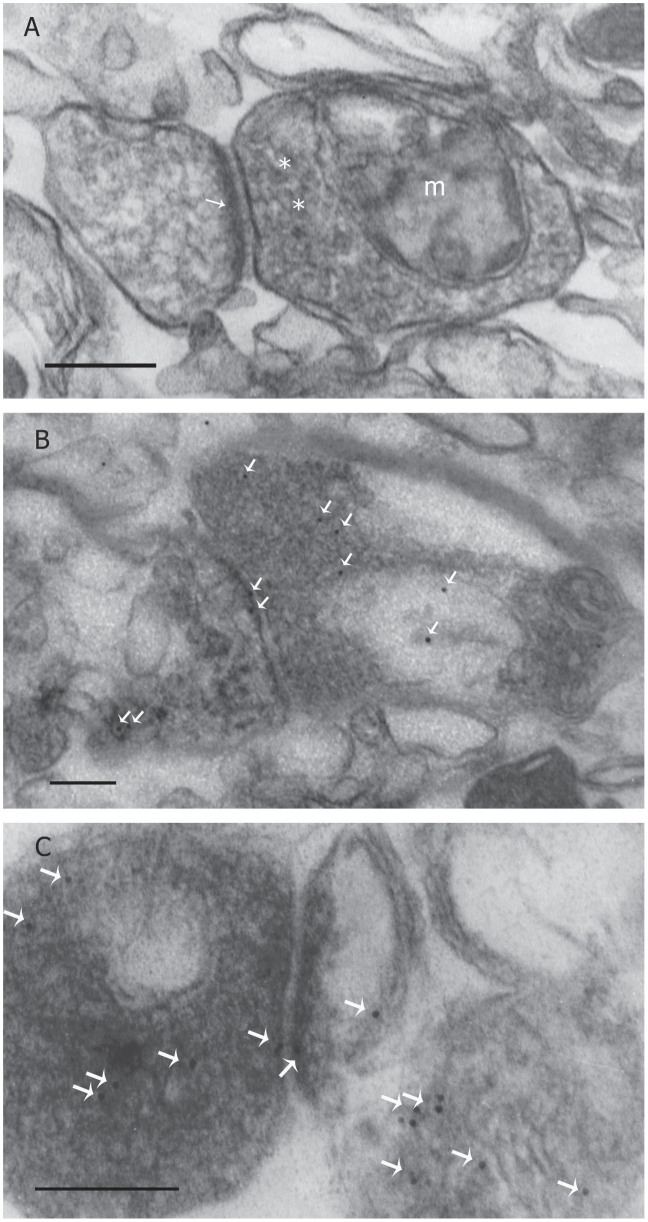
(A) Electron microscope analysis of a synaptosome. Synaptosomes from wild-type mice's cortex show a pre-synaptic element which contains mitochondria (m) and numerous synaptic vesicles (asterisk) and a post-synaptic element characterized by a synaptic junction with a postsynaptic density (arrows). (B,C) Mice cortex synaptosome immunolabelled with JNK2 antibody. Immunogold particles are visible and appear relatively numerous around vesicles of the pre-synaptic element and they are also diffused in the cytoplasm of the post-synaptic element (arrows). Scale bar: 200 nm.

The localization of JNK2 at the presynaptic site was also confirmed by electron microscopy technique. We evaluated the ultrastructure of synaptosomes by analyzing corticals from mice. The typical morphology of synaptosomes was clearly observed by electron microscopy (fig. 6A) and show mainly vesicles of 0.2–0.8 μm diameter that contain the complete presynaptic terminal (further distinguished by the conspicuous presence of neurotransmitter-filled vesicles, mitochondria, smooth endoplasmic reticulum, microtubules and neurofilaments); a synaptic cleft (about 20–30 nm); a postsynaptic component is distinguishable by presence of amorphous electrondense material containing black granules along the corresponding plasma membranes so-called ‘postsynaptic density’ (PSD)^9^.

Immunocytochemical analysis on synaptosomes shows the presence of JNK_2_ protein both in pre- and post-synaptic structure (fig. 6B).

### JNK2 colocalizes with presynaptic markers in mice cortical synaptosomes

**Figure 7:**
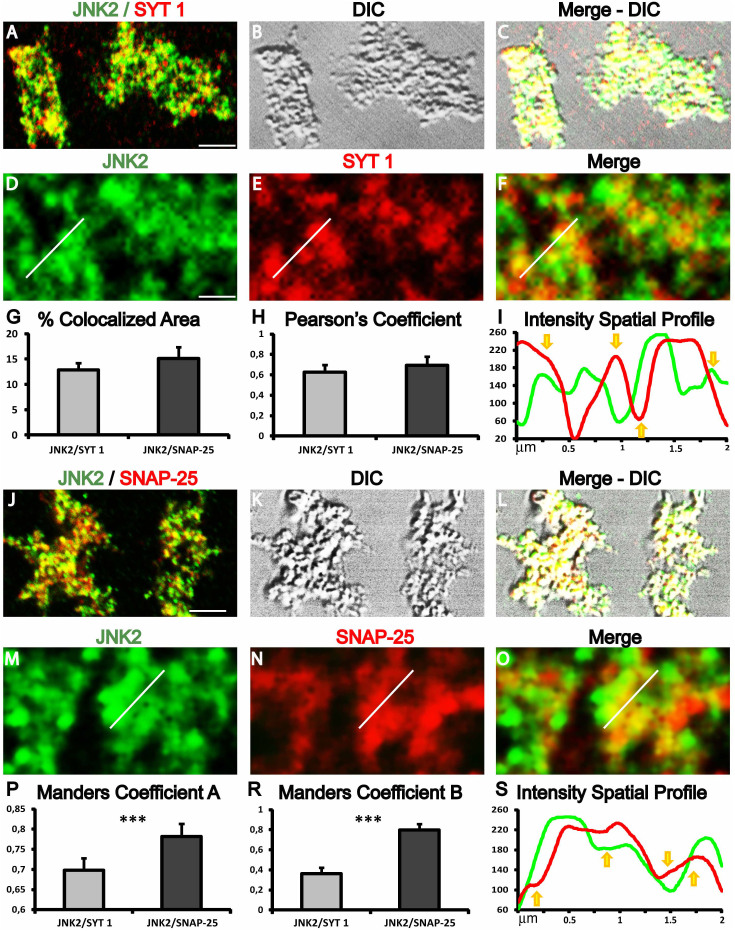
Synaptosomal JNK2 expression and colocalization with presynaptic markers. Confocal images and colocalization analysis of JNK2/SYT 1 (A–F) and JNK2/SNAP-25 (J–O) double labelling. (A) Fluorescence green/red channels merge of JNK2/SYT 1 double staining. (B) Transmitted light (DIC channel) view of JNK2/SYT 1 double staining. (C) Fluorescence and transmitted light channel merge. (D) JNK2 green channel immunofluorescence. (E) SYT 1 red channel immunofluorescence. (F) Fluorescence green/red channels merge of JNK2/SYT 1 double staining. (G) Histogram showing the percentages of colocalized area compared to the total immunofluorescent area. (H) Histogram showing Pearson's coefficients. (I) and (S) Traces of fluorescence intensity spatial profiles through the white line shown in D–E for JNK2/SYT 1 and in M–O for JNK2/SNAP-25 immunofluorescences. Yellow arrows indicate a qualitative visual evaluation of the correlative colocalization features. Arrows pointing up indicate a positive correlative colocalization (associated trend), while arrows pointing down display the absence of correlative colocalization (disjoint trend). (J) Fluorescence green/red channels merge of JNK2/SNAP-25 double staining. (K) Transmitted light (DIC channel) view of JNK2/SNAP-25 double staining. (L) Fluorescence and transmitted light channel merge. (M) JNK2 green channel immunofluorescence. (N) Snap25 red channel immunofluorescence. (O) Fluorescence green/red channels merge of JNK2/SNAP-25 double staining. (P) Histogram showing the percentages of Manders coefficient A. (R) Histogram showing Manders coefficient B. Data are derived from three independent experiments. In each experiment five microscopic fields for each experimental group were analyzed. Scale bar A and J = 6 μm; D and M = 0.7 μm.

Confocal microscopy experiments were performed to assess the colocalization of JNK2 with two well-known markers of the presynaptic machinery. Synaptotagmin-1 (SYT 1) is localized in synaptic vesicles and it triggers calcium-induced exocytosis^37^. Synaptosomal-associated protein 25 (SNAP-25) is a membrane protein with a cytosolic domain that contributes in the formation of the exocytotic fusion complex (SNARE complex), where it assembles with syntaxin-1 and synaptobrevin^38^. The double immunofluorescence staining for JNK2/SYN 1, (fig. 7A–F) or JNK2/SNAP-25 (fig. 7J–O) showed a high proportion of JNK2 immunoreactive synaptic puncta, indicating a widespread distribution of JNK2 while a complex colocalization pattern was characterized by different degrees of alternating single and double labeling in synaptosomal puncta. For quantitative analysis, to avoid interference from fluorescent dots aspecifically bound on the glass, image analysis was selectively performed by analyzing only the synaptosomal strucures identified through the transmitted light channel. Protein colocalization, including the evaluation of the colocalized threshold area, were analyzed by using two colocalization coefficients (Pearson's and Manders), and visual inspection of the spatial relations (Plot Profile) between the two immunoreactive fluorescence signal intensities. JNK2 colocalizes with both SYT 1 (13.00 ± 1.35%) and SNAP-25 (15.00 ± 2.21%) with respect to the total synaptosomal area (fig. 7G). The percentage difference (17%) was not significant suggesting that JNK2 does not preferentially colocalize with SYT 1 or SNAP-25. The Pearson's coefficient was 0.62 ± 0.07 and 0.69 ± 0.08 respectively, indicating that in all the synaptosomal structures JNK2 immunoreactivity colocalizes with both synaptosomal proteins (colocalization is considered positive for values ranging 0.5–1) (fig. 7H). Also in this case, the percentage difference was not significative (about 10%) suggesting that JNK2 does not correlate preferentially with SYT 1 or SNAP-25. Although Manders's coefficients are less efficient compared to Pearson's coefficient, for colocalization analysis, they can be used to evaluate the reciprocal association ratio between fluorescence markers. All Manders's coefficients were above 0.5 (association is considered positive for values ranging 0.5–1), with the exception of the JNK2 presence in the SYT 1 signal which was lower (0.36 ± 2.21) (fig. 7P–R). Interestingly, the percentual difference for both Manders coefficients were significatively higher for JNK2/SNAP-25 immunofluorescence respect to JNK2/SYT 1, (34% and 12%, respectively, *t*-test ***p < 0.001) suggesting a preferential interaction of JNK2 with SNAP-25. Plot profiles traces showed a rather heterogeneous fluorescence distribution for both JNK2/SYT 1 and JNK2/SNAP-25 double immunofluorescence (fig. 7I–S). Traces displaying associated spatial increases and decreases of fluorescence intensity (indicating colocalization; fig. 7 yellow arrows pointing up) were alternated to traces characterized by a disjoint trend (indicating lack of colocalization; fig. 7 yellow arrows pointing down). Associated and disjoint traces homogenously could be followed for about 0.5 micron which can be a reasonable estimation for a single presynaptic size (0.5–1.5 micron). This suggests that presynaptic structures display only colocalization or single staining for the analyzed markers.

### JNK2 isoform modulates presynaptic NMDA-dependent glutamate release

**Figure 8:**
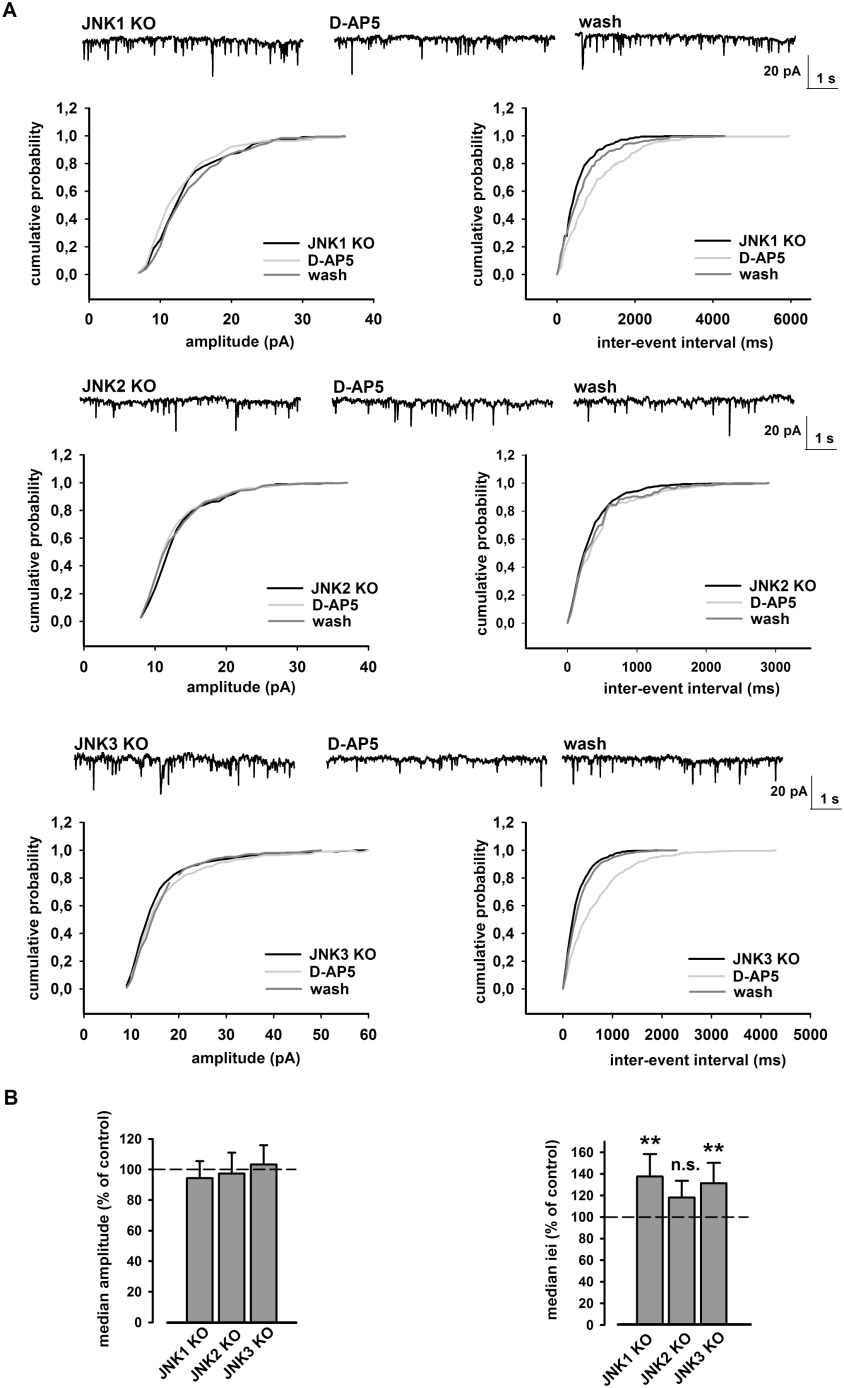
JNK2 isoform is involved in NMDA- mediated glutamate release. (A) Cumulative distributions of mEPSC amplitude (left) and iei (right) recorded from a single JNK isoform KO neuron in response to D-AP5 (50 μM). The traces on top are obtained from same neuron, in control conditions, during D-AP5 application and at D-AP5 washout. (B) Histograms of the median values expressed as percentage versus control (100%) of mEPSC amplitude (left) and iei (right) from n = 8 JNK1 KO, n = 7 JNK2 KO and n = 6 JNK3 KO neurons in response to D-AP5. **p < 0.01 (t-test).

Electrophysiological recordings were also performed in transgenic mice in order to confirm which JNK isoform was involved in NMDA-dependent glutamate release. For this, we recorded mEPSC from 8 neurons of JNK1 KO mice, 7 neurons of JNK2 KO mice and 6 neurons of JNK3 KO mice. We found that perfusion with D-AP5 induces an increase of the cumulative inter-event interval distribution in JNK1 KO (***p < 0.001, K-S test, JNK1 KO vs. D-AP5; fig. 8A) and JNK_3_ KO mice (***p < 0.001, K-S test, JNK3 KO vs. D-AP5; fig. 8A) and an increase of their median values (**p < 0.01, Student's t-test, control vs. JNK1 or JNK3 KO; fig. 8B). In both genotypes, the mEPSC amplitude was never affected by D-AP5 perfusion (*p* > 0.05, K-S test, JNK1 or JNK3 KO vs. D-AP5; fig. 8A; *p* > 0.05, Student t-test, control vs. JNK1 or JNK3 KO; fig. 8B). Notably, the decrease of glutamate release due to D-AP5 was specifically prevented in JNK2 KO mice. Indeed, perfusion with D-AP5 did not produce significant changes neither in mEPSC iei (*p* > 0.05, K-S test, JNK2 KO vs. D-AP5; fig. 8A; *p* > 0.05, Student's t-test, control vs. JNK2 KO; fig. 8B) nor in amplitude (*p* > 0.05, K-S test, JNK2 KO vs. D-AP5; fig. 8A; *p* > 0.05, Student's t-test, control vs. JNK2 KO; fig. 8B). Overall these electrophysiological data suggest that only the JNK2 isoform mediates presynaptic release of glutamate under our experimental conditions.

## Discussion

Here we report that JNK2 protein is implicated in the control of the presynaptic NMDA-evoked glutamate release in cortex. JNK has been widely demonstrated to be downstream target of postsynaptic NMDA receptors^39^. Importantly, the NMDAR activity is linked to the presence of JNK interacting protein 1 (JIP1) protein^28^. Therefore this intracellular signaling pathway involves the combined interaction of three partners: NMDAR, JIP1 and JNK. Although there is general consensus that NMDA receptors are located only at the post-synaptic site, few are the evidences that NMDAR are also present at the presynaptic site where they control neurotransmitter release^26^,^40^. However it has never been directly demonstrated, at the presynaptic site, that the NMDAR activity is mediated by the interaction with JIP1 and JNK. To date, only few works have proposed a presynaptic role for JNK. In PC12 cells it has been shown that JNK, activated by nerve growth factor (NGF), controls Ca^2+^-evoked release due to direct phosphorylation of synaptotagmin-4 (SYN4)^41^. However, this protein is known to be located either pre or postsynaptically. Recent findings suggested that JNK can be stimulated by NGF via presynaptic p75^NTR^ receptor^16^. Furthermore, JNK3 has been shown to be involved in the molecular organization of the presynapse^42^. Using presynaptic release assay, together with biochemical, imaging and electrophysiological experiments, here we identify for the first time that JNK is also present at presynaptic sites. Specifically we defined that JNK plays an essential and specific role in controlling glutamate release mediated by presynaptic NMDAR in cortical nerve endings. Release experiments were performed on synaptosomes in a superfusion system^13^ where each event affecting glutamate release is attributable only to the functional presynaptic compartment. To inhibit JNK protein activity we have used a commercial cell-permeable peptide (L-JNKi1) containing an effector part, JNK binding domain (JBD), linked to a cargo sequence, i.e. the 10 amino acid long arginine rich domain of the HIV-1 Tat protein (48–57). The effect of L-JNKi1 at the presynaptic compartment could be justified since we have demonstrated, for the first time, the presynaptic expression of both JIP1 and JNK by using a synaptic fractionation protocol.

Our biochemical experiments on isolated cortical presynaptic terminals show that, among the applied stimuli, only NMDA induces JNK phosphorylation. This effect was slightly but not significantly reduced by L-JNKi1 since, as already published for D-JNKi1^43^,^44^, L-JNKi1 does not block JNK activation but its downstream effect. In addition, using synaptosomes fractionation analysis, we showed that NMDA stimulus increases JNK phosphorylation at the presynaptic fraction, confirming the presynaptic activation of JNK.

L-JNKi1 prevented the NMDA-, but nor the KCl- or AMPA-evoked glutamate release. This peptide competes with the JNK binding domain (JBD) disrupting the interaction between JIP1 and JNK, consequently the downstream activity of JNK is blocked^31^. Our data strongly suggest that presynaptic JNK signaling pathway might be selectively engaged following NMDA receptor activation. This observation is further supported by the measurement of vesicle exocytosis by using a well established method^45^,^46^. By means of a fluorescent dye (Synaptogreen) preloaded in presynaptic vesicle, it is possible to follow the decrease of fluorescence when the dye is released in the extracellular space. We observed a reduction in fluorescence intensity during exposure to NMDA which was prevented by L-JNKi1, indicating once again that presynaptic JNK controls NMDA-evoked release. The peptide effect is not imputable to the Tat-containing part that does not produce any effect on glutamate release. In fact, either HIV-1 Tat or 20-mer Tat (41–60) show a potentiating effect of the NMDA responses^47^,^48^, while smaller Tat peptide (41–60) augmented acetylcholine release^49^. In line with these findings, here we show that Tat (41–60) and Tat (31–50), containing the 48–57 aa sequence, did not affect glutamate release.

An additional experiment to evaluate the exocytosis in cortical synaptosomes has been done using time-lapse imaging. Among the stimuli used only NMDA-evoked Synaptogreen exocytosis was reduced by L-JNKi1 application which corroborate our previous results. In addition, also our electrophysiological findings conducted in mouse cortical slices revealed a functional role of JNK in mediating the NMDA-induced glutamate release.

In support of our data a more stable form of L-JNKi1, D-JNKi1, reduced excitotoxicity and protected against cerebral ischemia^44^,^50^, and albeit only postsynaptic activity was studied, a presynaptic effect cannot be ruled out.

In order to identify which JNK isoform is involved in neurotransmitter release during NMDA stimulation we also employed JNK transgenic knock-out mice^21^. JNK3 deletion leads to protection from excitotoxicity^51^ and hypoxia^52^, while JNK1 and JNK2 seem to be important for apoptosis during brain development^19^. Using knockout mice for single JNK isoforms, we report that JNK2 is the critical player involved in this presynaptic phenomenon. Notably JNK2 KO mice display a dramatic reduction of NMDA-evoked release, being associated with a decreased NMDA-elicited JNK activation, as demonstrated by western blot experiments.

Electron microscopy experiments were performed on cortical synaptosomes and, as previously reported^9^, the synaptosomal morphology was well preserved. Whereas JNK1^53^ and JNK3^42^ have been associated with a synaptic presence and function, on the other hand JNK2 was only reported to be localized between the cytoplasm and the nucleus^54^. Here, by immunogold staining, we have clearly identified the presence of JNK2 at both post- and pre-synaptic site. These results were confirmed and extended in confocal microscopy where a quantitative approach allowed to get more information of JNK 2 expression pattern. Colocalization experiments indicate that JNK2 is expressed in synaptic puncta in high proportion. This observation supports our functional data demonstrating that JNK2 can interfere with the SNARE complex machinery thereby controlling glutamate release. Finally, also our electrophysiological recordings highlight that JNK2 KO mice display reduced miniature EPSCs attributable to activation of presynaptic NMDA receptors.

Altogether our data led us to conclude that JNK is also localized at presynaptic site being able to control glutamate release evoked by NMDA stimulus.

We here describe that specifically the isoform 2 of JNK cooperates with NMDA receptors in regulating glutamate release. In the experiment in which the synaptic exocytosis was measured, the inhibition of JNK activity reduces neurotransmitter release when NMDA is applied. This leads to speculate that the mechanism by which NMDA controls glutamate overflow might pass through an interaction between JNK2, presynaptic JIP1 and other proteins of the release machinery. This aspect needs further investigation in future work.

In light of the mounting evidence indicating the involvement of JNK signaling in different diseases^55^, the present findings highlight a presynaptic localization and function of JNK2. This protein will represent a novel and selective target to modulate release-regulating NMDA autoreceptors, whose activation reinforces glutamate release from the presynaptic component of asymmetric synapses then controlling excitatory transmission in CNS.

## Methods

### Animals and brain tissue preparation

C57BL6/J and JNK knock-out (KO) (JNK1 – JNK2 – JNK3)^21^ mice, 5 months old male and female, were killed by decapitation in accordance with the guidelines established by the European Communities Council (Directive 2010/63/EU of 22 September 2010) and accepted by the Italian Ministry of Health and approved by the Ethical Committee on animal experiments of EBRI “Rita Levi-Montalcini” Foundation (Rome, Italy). Brains were rapidly dissected out on ice in order to collect cortical tissues. All the procedures necessary to prepare the brain fractions were done at 0–4°C.

### JNK knock-out animals

Adult male and female of transgenic JNK KO (JNK1 – JNK2 – JNK3) mice were obtained by R.J. Davis from University of Massachusetts. They have been generated by knocking down each member of the JNK family (JNK1, Ref. 35; JNK2, Ref. 56; JNK3, Ref. 51) and maintained as homozygotes on a C57BL/6 genetic background.

### Synaptosome preparation

Purified synaptosomes were prepared as previously described^11^,^13^,^57^. Briefly, the tissue was homogenized in 10 volumes of 0.32 M sucrose, buffered to pH 7.4 with Tris-(hydroxymethyl)-amino methane [Tris, final concentration (f.c.) 0.01 M] using a glass/Teflon tissue grinder (clearance 0.25 mm); the homogenate was centrifuged at 1,000 × g for 5 min to remove nuclei and debris and the supernatant was gently stratified on a discontinuous Percoll (Sigma Aldrich, Italy) gradient (2%, 6%, 10% and 20% v/v in Tris-buffered sucrose) and centrifuged at 33,500 × g for 5 min. The layer between 10% and 20% Percoll (synaptosomal fraction) was collected and washed by centrifugation. The synaptosomal pellets were resuspended in a physiological solution (Standard Medium) with the following composition (mM): NaCl, 140; KCl, 3; MgSO_4_, 1.2; CaCl_2_, 1.2; NaH_2_PO_4_, 1.2; NaHCO_3_, 5; HEPES, 10; glucose, 10; pH 7.2–7.4.

### Synaptosomal glutamate release

Cortical synaptosomes were incubated for 45 min at 37°C in a rotary water bath in the presence of [^3^H]D-aspartate ([^3^H]D-ASP (final concentration 50 nM). When indicated, L-JNKi1 (2 μM) was added in the incubation medium for 30 min. After the labeling period, synaptosomes were maintained and superfused with physiological solution at 37°C layered in a Superfusion System (Ugo Basile, Comerio, Varese, Italy) for 48 min.

After 36 min of re-equilibration period, four consecutive 3 min fractions (termed b1 to b4) were collected. N-Methyl-D-aspartic acid (NMDA) (100 μM) and glycine (1 μM), (S)-α-Amino-3-hydroxy-5-methyl-4-isoxazolepropionic acid ((S) AMPA) (50 μM), or (HIV-1)-encoded transactivator of transcription (Tat) 41–61 (2 nM) and Tat 31–51 (2 nM) were applied at the end of the first fraction collected (b1) and maintained in the superfusion medium till the end of the superfusion period. In NMDA experiments the medium was replaced, at *t* = 20 min, with a Mg^2+^-free ions solution to permit NMDAR activation. In KCl experiments synaptosomes were transiently (90 s) exposed, at *t* = 39 min, to high K^+^ containing medium (8 mM). In these experiments, fractions were collected according to the following scheme: two 3 min fractions (basal release), one before (*t* = 36–39 min) and one after (*t* = 45–48 min) a 6 min fraction (*t* = 39–45 min; evoked release). Fractions collected and superfused synaptosomes were counted for radioactivity. The amount of radioactivity released into each superfusate fraction was expressed as a percentage of the total synaptosomal tritium content at the start of the fraction collected (fractional efflux). Agonist and high K^+^-induced effects were estimated by subtracting the neurotransmitter content into the fractions corresponding to the basal release from those corresponding to the evoked release.

### Measurements of synaptic vesicle exocytosis

#### Spectrophotometer measurements

Synaptogreen (SIGMA-ALDRICH, Italy), also known as FM1-43, was used to measure synaptic vesicle fusion with the plasma membrane as previously described^45^,^46^. In brief, synaptosomes (0.3 mg/mL) were incubated in 2 ml of Standard Medium for 2 min at 37°C in mild agitation. Synaptogreen (50 μM) was added and after 1 min stimulation with NMDA (100 μM) and glycine (1 μM) was applied to load the fluorescent dye. After 3 min, synaptosomes were washed twice to remove non-internalized Synaptogreen in Standard Medium containing 1 mg/mL BSA. Synaptosomes (200 μg of proteins) were then resuspended in 2 mL of Mg^2+^-free Standard Medium, and incubated in a stirred and thermostated cuvette maintained at 37°C in a RF-5301PC spectrofluorophotometer (Shimadzu, Japan). Release of accumulated Synaptogreen was induced by the addition of NMDA (100 μM) and glycine (1 μM) after 10 min of re-equilibration, and measured as the decrease in fluorescence upon release of the dye into solution (excitation 510 nm, emission 625 nm) for 6 min after stimulus. Data points were obtained at 2 s intervals, and data presented as the Ca^2+^-dependent decrease in FM1-43 fluorescence. The synaptosomes were pre-incubated, when indicated, with L-JNKi1(Sigma-Aldrich, USA) for 30 min before depolarization with NMDA and glycine. The average of the fluorescence of the last 20 registered points are compared to the one at the stimulus point. The value is expressed as percentage of decreased fluorescence after stimulus for each trace.

#### Microscopy measurements

Synaptosomes were seeded on coverslips in Standard Medium for staining procedures. FM1-43 loading into synaptic vesicles was performed as described above for spectrophotometer measurements and placed on a time-lapse system mounted on an inverted fluorescence microscope (TiE; Nikon, Japan), equipped with an incubation chamber (Okolab), a cooled CCD camera (Clara; Andor), Perfect Focus System to avoid z-axis focus fluctuations and a Niss Elements imaging software (Nikon). Video recordings were performed with a 40 × oil objective (N.A 1.4) for 6 (30 mM KCl) or 30 min (NMDA 100 μM + glycine 1 μM) by taking 14 bit images every 2 s at room temperature (25°C). Fluorescence was recorded and video images were analyzed by using the Spot module of the ImarisSuite 7.6® software (Bitplane A.G., Zurich, Switzerland) by selecting a 1.5 μm radius diameter of fluorescent puncta. Fluorescence intensity was measured in 15–20 randomly selected synaptosomes within the microscopic field. The criteria for fluorescence puncta inclusion in the data analysis were the spherical shape ranging between 0.5–1.5 micron and the stimulation-dependent destaining response. Destaining time courses were generated by normalization of each fluorescence spot trace. Quantification of FM1-43 responses was accomplished by calculating the average percentage of fluorescence loss. A group of control experiments was run to test the specificity of Synaptogreen loading and destaining. Ca^2+^ free medium drastically reduced the dye loading and inhibited the stimulus-induced dye release. The Synaptogreen photobleacing rate was also measured in absence of the stimulus and compared to the stimulated Synaptogreen destaining traces for each experimental group.

### Stimulation of synaptosomes in biochemical experiments

Stimulation of synaptosomes was performed in batch as previously reported^27^. Re-suspended synaptosomes were divided into 0.2 ml aliquots and gently agitated at 37°C until stimulation. Following 20 min mild agitation at 37°C, JNK inhibitor L-JNKi1 (2 μM) or NMDA receptor inhibitor D-AP5 (50 μM) were added as indicated and left for 30 min at 37°C before stimulus and till the end of the experiments. Synaptosomal suspensions were subjected to differential stimulation (see Results for details) with either NMDA (100 μM) + glycine (1 μM) or AMPA (100 μM) for 10 min at 37°C. KCl (8 mM) stimulus was added for 90 seconds after which 0.2 ml of warm medium containing inhibitors, where needed, was added.

The experiments were terminated by adding 0.6 ml of cold HEPES buffer and immediate centrifugation at 12,000 × *g* to pellet the synaptosomes. In NMDA stimulation experiments, external Mg^2+^ ions were omitted to avoid the functional inactivation of the receptor.

The final pellets were lysed in ~100 μl of Lysis Buffer solution (LB) made up of 1% Triton X-100 (Serva, Germany), complete protease inhibitor cocktail solution (Serva, Germany), phosphatase inhibitor cocktail solution (Serva, Germany) and the following components (mM): TRIS acetate, 20; sucrose, 0.27; EDTA, 1; EGTA, 1; Na Orthovanadate, 1; NaF, 50; Na Pyrophosphate, 5; Na β-glycerophosphate, 10; DTT, 1.

Protein concentrations were then determined by Bradford assay and the samples were directly analyzed by immunoblotting following resuspension in Laemmli buffer.

### Ultrastructural fractionation

As reported in Ref. 9, after stimuli or just after preparation, synaptosomes were pelleted and resuspended in 300 μL HEPES buffer (4°C; pH 7.4) and 50 μL was removed and kept as a total. Synaptosomes were pelleted by centrifugation (16 000 g; 5 min; 4°C) and resuspended in 300 μL 0.32 m sucrose and 0.1 mm CaCl_2_. Protease inhibitors were used in all extraction steps. Synaptosomal solution was further diluted 1:10 in ice-cold 0.1 mm CaCl_2_ and then mixed with an equal volume of 2 × solubilisation buffer (2% Triton X-100, 40 mm Tris, pH 6.0; 4°C). Following 30 min incubation at 4°C the insoluble material (synaptic junctions) was pelleted by centrifugation (40 000 g, 30 min, 4°C). The supernatant (NSSP) was decanted and proteins precipitated with 6 volumes of acetone at −20°C and centrifuged (18 000 g; 30 min; –15°C). The synaptic junction pellet was resuspended in 10 volumes of 1 × solubilisation buffer (1% Triton X-100, 20 mm Tris, pH 8.0; 4°C) and incubated for 30 min at 4°C and then centrifuged (40 000 g, 30 min, 4°C). The pellet contained the insoluble postsynaptic density and the supernatant contained the presynaptic active zone. The protein in the supernatant (presynaptic fraction) was acetone precipitated and collected as above. Proteins were then treated for Western Blot (WB) analysis.

### Immunofluorescence

Immediately after preparation synaptosomes were seeded on coverslip coated with Poly-L-Lysine (50 μg/ml), washed twice with PBS and fixed in 2% (w/v) freshly prepared paraformaldehyde in PBS for 10 min at room temperature. After four washes synaptosomes were permeabilized with 0.05% (v/v) Triton X-100/PBS for 5 min, washed and incubated overnight in a mix solution of the following primary antibodies: rabbit anti-JNK2 (Santa Cruz, USA) and mouse anti-Synaptotagmin I (Santa Cruz, USA), or rabbit anti-JNK2 and mouse anti-SNAP25 (SMI 81) (Covance, USA). All primary antibodies were diluted 1:200 in 0.05% (v/v) Triton X-100/PBS at 4°C. Unbound antibody was removed by four washes 10 min each with PBS at room temperature. Bound antibody was detected by incubation with donkey anti-rabbit Alexa 488 (1:500) and donkey anti-mouse Alexa 555 (1:600) conjugated secondary antibodies (Invitrogen, USA) at 4°C for 2 h. After three washes with PBS at room temperature, coverslips were mounted on slides with Hydromount™ mounting gel. Controls were performed by omitting the primary antibodies.

### Confocal microscopy and image analysis

Slides were examined under a confocal laser scanning microscope (Leica SP5, Leica Microsystems, Wetzlar, Germany) equipped with 4 laser lines and a transmitted light detector for differential interference contrast (DIC; Nomarski) imaging. Confocal acquisition setting was identical between slides. Bright field images of the synaptosomes were corrected for homogeneities of the brightness across the image by using Adobe Photoshop high-pass filter. Contrast of synaptosome edges was enhanced with ImageJ CLAHE (Contrast Limited Adaptive Histogram Equalization; http://rsbweb.nih.gov/ij/plugins/clahe/index.html) filter plugin. For production of figures, brightness and contrast of images were globally enhanced by using linear histogram correction and images were slightly oversaturated. Image analysis was performed by using Imaris Suite 7.4® or Image J 1.4 (imagej.nih.gov/ij/) softwares on seven different images derived from each group. Image analysis was performed under visual control to determine thresholds that subtracts background noise and takes into account synaptosomes structures. For all image-processing steps, the images were compared with the original raw data to make sure that no structures were introduced that were not seen in the original data series or that structures present in the original data series were not removed. To determine fluorescent signal colocalization between different channels the ImarisColoc module was used. Before colocalization analysis, a median filter was applied to the images to reduce the background noise and source channels threshold were established by automatic thresholding of the P Value. The degree of channels colocalization was analyzed by considering the following indexes: the % of dataset colocalized (colocalized area), thresholded Manders's coefficient A and B, Pearson's coefficient in colocalized area. To evaluate the amount of the total synaptosomoal area several masks, by using Imaris Surface module, were manually drawn enclosing the entire synaptosomes visualized through the light transmitted channel. Inside these masks, to evaluate the relative immunofluorescent areas a mask for each channel was automatically drawn using the Imaris Surface module. To evaluate the spatial relations between channels intensity we used the ImageJ tool Plot Profile to create a plot of fluorescence intensity values across a line drawn on the image.

### Samples preparation for electron microscopy

Synaptosomal fraction was fixed in 2.5% glutaraldehyde (Electron Microscopy Science-EMS) in 0.05 M phosphate buffer pH 7.4 for 3 hours at room temperature, post-fixed in 1% osmium tetroxide (Electron Microscopy Science-EMS), dehydrated in graded acetone and embedded in Spurr (Epoxy resin, EMS). For a morphological analysis, semithin sections (1 μm thick) were cut on a Reichert-Jung Ultracut ultramicrotome and stained with toluidine blue. Ultrathin sections were cut and stained with uranyl acetate and lead citrate, then examined with a Zeiss electron microscope.

For post-embedding immunolabeling, sections (80 nm) were placed on 200-mesh formvar carbon nickel grids (EMS), etched in 1% sodium periodate for 60 min and in H2O2 1% for 15 min. Residual aldehyde groups were quenched with 0.05 M glycine in PBS, pH 4 for 20 min. After 30 minutes incubation in blocking buffer (phosphate buffer solution pH 7,4 added with 5% bovine serum albumin), the samples were incubated with polyclonal anti-JNK2 antibody (1:20; SC-827 NH2-terminal S. Cruz) in phosphate-buffered saline added with 1% bovine serum albumin (incubation buffer) overnight at room temperature. After washing, samples were incubated in incubation buffer with goat anti-rabbit secondary antibody conjugated to 10-nm gold particles (1:10; AURION Immuno Gold Reagents & Accessories, Wageningen, the Netherlands) for 3 hours at room temperature. Samples were washed in incubation buffer, then in distilled water and counterstained with saturated uranyl acetate solution in H_2_O for 1 hour. Grids were air-dried and viewed under the electron microscope.

### Electrophysiology

C57BL6/J or JNK KO mice (2–3 months old) were anesthetized with halothane and killed by decapitation. The brain was rapidly removed from the skull, and combined entorhinal-hippocampal slice were cut (250 μm) in cold artificial cerebrospinal fluid (ACSF) using a vibratome, according to the method described by Jones and Heinemann^58^. ACSF composition was (mM): NaCl, 124; KCl, 3.0; MgCl_2_, 1.0; CaCl_2_, 2.0; NaH_2_PO_4_, 1.25; NaHCO_3_, 26; glucose, 10; saturated with 95% O_2_, 5% CO_2_ (pH 7.4). After an incubation period of 1 h in ACSF at 33.5°C, slices were maintained at room temperature or transferred to a submerged recording chamber (2.5–3 ml min-1, 33.5°C), on the stage of an upright microscope (Eclipse FN1, Nikon, Italy), equipped for infrared video microscopy (Hamamatsu, Japan).

Layer II neurons of the EC were recorded in whole-cell patch-clamp configuration using 1.5 mm borosilicate glass electrodes (3–4 MΩ) pulled with a vertical puller (PP-83 Narishige, Japan) in voltage-clamp mode^59^. Filling solution containing (in mM): CsMeSO_4_, 130; HEPES, 5; EGTA, 0.5; MgCl_2_, 1; NaCl, 1; CaCl_2_, 0.34; QX-314, 5 and MK-801, 1; adjusted to pH 7.3 with CsOH. Unless otherwise stated, neurons were held at −70 mV. Current signals were filtered at 2 kHz and digitized at 10 kHz using a Multiclamp 700 B differential amplifier linked to a Digidata 1440A, operated by the pClamp10 software (Molecular Devices, USA). No series resistance compensation was implemented, in order to keep a low signal-to-noise ratio, however, series resistance and whole-cell capacitance were monitored continuously during the experiment and recordings were discarded if series resistance changed by more than 15% from control conditions. Spontaneous synaptic events were analyzed offline using the pClamp10 software package (Molecular Devices, USA). The spontaneous events were detected from 3 min trace records, through an algorithm based on the minimization of the sum of squared errors between data and a template function. Single template waveforms were created by averaging a number of spontaneous events following a visual inspection of a representative trace in each cell. The template-matching threshold was set between 5 and 5.5, providing a good balance to avoid detection of false events.

### Western Blot

Equal amount of proteins (~15 μg for each condition) were resolved by 10% SDS-polyacrylamide gels and blotted onto PVDF membrane (Serva, Germany). The resulting blot was blocked for 1 h at room temperature using Tris-buffered saline-Tween (t-TBS) (M) Tris, 0.02; NaCl, 0.15; Tween 20, 0.1%) containing 5% skimmed milk. The membranes were then incubated overnight at 4°C with specific antibodies: rabbit phospho-SAPK/JNK (Thr 183/Tyr185) (p-JNK) 1:500 (Cell Signaling, USA) this antibody recognizes altogether the phosphorylated bands (46 and 54 kDa) of JNK1, JNK2 and JNK3, rabbit SAPK/JNK 1:1000 (Millipore, Italy) this antibody recognizes altogether the bands (46 and 54 kDa) of JNK1, JNK2 and JNK3, mouse β-actin 1:30000 (Sigma Aldrich, Italy), mouse anti NR1 1:1000 (Millipore), mouse anti NR2a and NR2b 1:1000 (Millipore), rabbit anti GluA1 and GluA2 1:1000 (Millipore).

After 50 min of wash in t-TBS, the blots were incubated for 1 h at room temperature with peroxidase-conjugated goat anti-rabbit or anti-mouse IgG secondary antibodies (UCS Diagnostic).

After 50 min of washes in t-TBS bands immunoreactivity was detected by enhanced chemiluminescence (ECL; WESTAR, Cyanagen, Italy). In all experiments where an evaluation of phosphorylated proteins was required, pan antibodies were applied on the same membranes after stripping procedure (stripping buffer from SignaGen, USA).

### Statistical Analysis

In release experiments, analysis of variance was performed by ANOVA followed by Newman-Keuls multiple-comparison test, when comparing only a single treatment group a Student's two-tailed t-test was used. Data were considered significant for *p* < 0.05 at least. Appropriate controls with enzyme inhibitors were always run in parallel.

Statistical analysis for biochemical experiments was performed with GraphPad PRISM 4 (GraphPad Software, USA). Values shown represent the mean ± SEM of a different number of separate experiments (as indicated in figure legends). One-way or Two-ways ANOVA followed by Tukey's test was carried out when intergroup comparisons were required.

Changes in miniatures events amplitude or inter-event interval was determined by comparison of their cumulative distributions, with the Kolmogorov-Smirnov (K-S) test, or by comparison of their median values, using the 2-tailed Student t-test. The threshold for statistical significance was set at p < 0.05.

## Author Contributions

Conceived and designed the experiments: M.F. Performed the experiments: M.F., F.F., D.M., C.F., M.G., S.D.P., A.N. and S.S. Analyzed the data: M.F., F.F., D.M. and S.S. Contributed reagents/materials/analysis/funds tools: M. Marchi, N.B.M., M.Morbin, M.D., A.P., R.J.D. and R.N. Wrote the paper: M.F. and R.N.

## Supplementary Material

Supplementary Information Supplementary Information
