# Supplementary material for: Presynaptic c-Jun N-terminal Kinase 2 regulates NMDA receptor-dependent glutamate release
Source: Sci Rep. 2015 Mar 12;5:9035. doi: 10.1038/srep09035 (PMC4357012; doi:10.1038/srep09035)

Supplementary Information

Title:
Presynaptic c-Jun N-terminal Kinase 2 regulates NMDA receptor-dependent glutamate release

Robert Nisticò[1,8], Fulvio Florenzano [1], Dalila Mango [1,2], Caterina Ferraina [1], Massimo Grilli [3], Silvia Di Prisco [3], Annalisa Nobili [2,4], Stefania Saccucci [5], Marcello D'Amelio [2,4], Michela Morbin [5], Mario Marchi [3,6], Nicola B. Mercuri [2], Roger J. Davis [7], Anna Pittaluga [3,6] and*Marco Feligioni [1]

Supplementary Figure 1. Presynaptic proteins and receptor subunit level during NMDA and L-JNKi1 treatment.

A) Representative western blot showing unchanged NMDA and AMPA receptor subunits protein levels upon NMDA (100 μM) + glycine (1 μM) and L-JNKi1(2 μM) treatment in wild-type mice cortical synaptosomes. B) Representative Western blot showing unchanged presynaptic protein, syntaxin 1a (STX1a), synapsin (SYN), MUNC18-1, SYT 1, protein levels upon NMDA and L-JNKi1 treatment in wild-type mice cortical synaptosomes. C) Representative Western blot showing JNK protein expression in JNK KO mice cortical synaptosomes. Anti-JNK shows the bands corresponding to JNK1 (46 and 54 kDa), JNK2 (46 and 54 kDa) and JNK3 (54 kDa).
WB shown are cropped and representative.

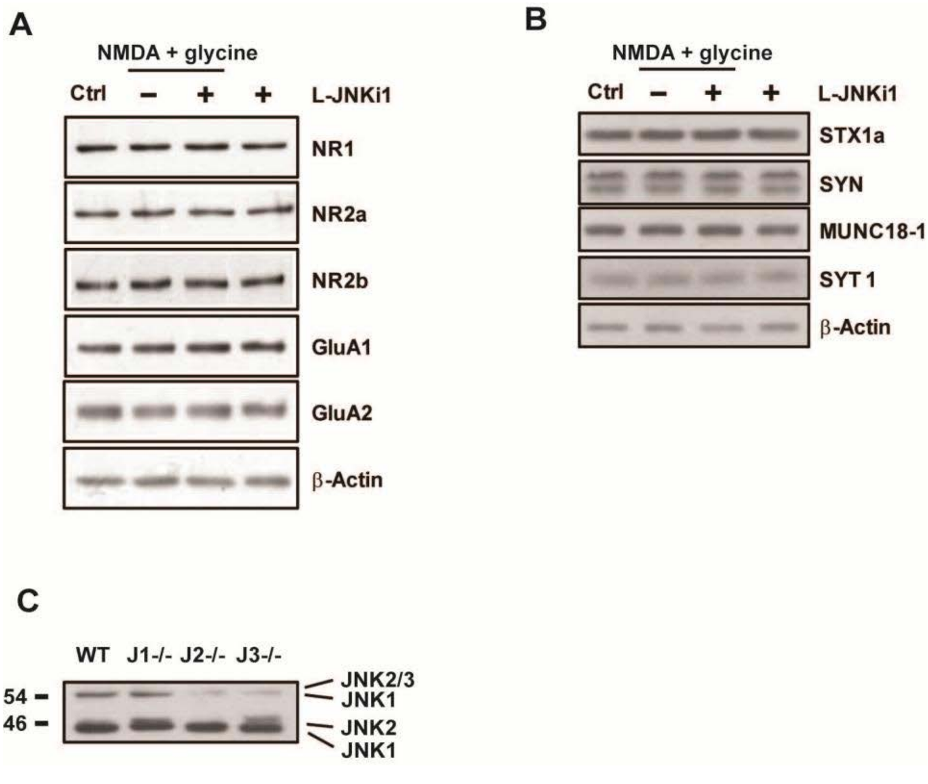

Supplement: Supplementary Information [file srep09035-s1.pdf]
